# Delayed management of acute type A aortic dissection with concomitant coronary artery bypass graft

**DOI:** 10.1186/s13019-024-02821-9

**Published:** 2024-06-05

**Authors:** Zohaib R. Khawaja, Yusuf M. Aboutabl, Gabriel E. Cambronero, Robert G. Willis, Aaron S. Gilani, Bartlomiej R. Imielski

**Affiliations:** Department of Cardiothoracic Surgery, Wake Forest University School of Medicine, Winston-Salem, NC USA

**Keywords:** Aortic dissection, Coronary artery bypass graft, Coronary angiography

## Abstract

**Background:**

Pre-operative coronary angiography and concomitant, planned coronary artery bypass are infrequently performed with type A aortic dissection repair. We present a case in which pre-operative coronary computed tomography angiography was appropriate, and subsequent dissection repair and concomitant coronary artery bypass were successfully performed.

**Case Presentation:**

The patient is a 58-year-old male with heart failure with preserved ejection fraction, renal insufficiency, hypertension, obesity, and smoking history, who presented with a three-to-four-day history of persistent back pain, worsening exertional dyspnea, and orthopnea, as well as a two-to-three month history of dyspnea, lower extremity edema, and intermittent angina. He was diagnosed with an acute type A aortic dissection and anti-impulse control was initiated. However, repair was delayed in order to allow apixaban to metabolize and decrease the risk of bleeding, as the patient was approximately six days post-dissection, without malperfusion, with a well-controlled blood pressure on anti-impulse therapy, and had received five days of anticoagulation. During this time, coronary computed tomography angiography was performed to assess the need for concomitant revascularization and showed coronary artery disease. Ascending aorta hemiarch replacement with aortic valve resuspension, two-vessel coronary artery bypass grafting, and left atrial appendage clipping were performed successfully.

**Conclusions:**

Pre-operative imaging can be considered in a select group of acute type A aortic dissections that present without malperfusion, and with well-controlled blood pressure on anti-impulse/negative inotropic therapy.

## Background

Given the mortality associated with acute type A aortic dissections (TAAD), the use of coronary angiography to assess for coronary artery disease (CAD) prior to aortic repair is limited and controversial. A general consensus states that coronary angiography should be avoided pre-operatively due to delay of surgical intervention, risk of aortic rupture or exacerbating malperfusion, and its relatively low rate of detection [1]. Additionally, prophylactic coronary artery bypass grafting (CABG) is typically avoided for several reasons, including unnecessarily exposing patients to the risks associated with CABG, unknown target artery anatomy, and questionable internal mammary artery inflow [1]. Thus, planned CABG is infrequently performed concomitantly with TAAD repairs. However, one study found that 34.8% and 42.9% of patients with acute and chronic TAAD, respectively, who underwent pre-operative coronary angiography had one or more lesions of greater than 50% narrowing.^2^ The same study found no fatal complications were associated with pre-operative coronary angiography and concluded it should be performed in stable patients with acute TAAD and in all patients with chronic TAAD [2]. Here, we present a case in which a pre-operative coronary computed tomography angiography (CCTA) was performed prior to TAAD repair and concomitant CABG.

## Case Presentation

A 58-year-old male smoker with known heart failure with preserved ejection fraction, renal insufficiency, hypertension, and obesity presented with a two-to-three month history of dyspnea, lower extremity edema, and intermittent angina. He was admitted to a referring hospital with a three-to-four-day history of persistent back pain, worsening exertional dyspnea, and orthopnea. He had mild troponinemia and an elevated B-type natriuretic peptide. Chest plain-film radiography demonstrated cardiomegaly, vascular congestion, and pleural effusion. Electrocardiogram showed new-onset atrial fibrillation with rapid ventricular response, and he was started on apixaban. He was worked up for an acute-on-chronic heart failure exacerbation and underwent a myocardial perfusion imaging showing a large anterior septal and apical region scar without reversibility. This was consistent with a former myocardial infarction in the left anterior descending (LAD) artery territory, and the patient corroborated that years ago he had an episode of severe chest pain radiating to the right shoulder which left him bedridden. A transesophageal echocardiogram for cardioversion was performed, which diagnosed an acute TAAD and demonstrated an ejection fraction of 30%. He was initiated on anti-impulse control and promptly transferred to our facility.

**Fig. 1:**
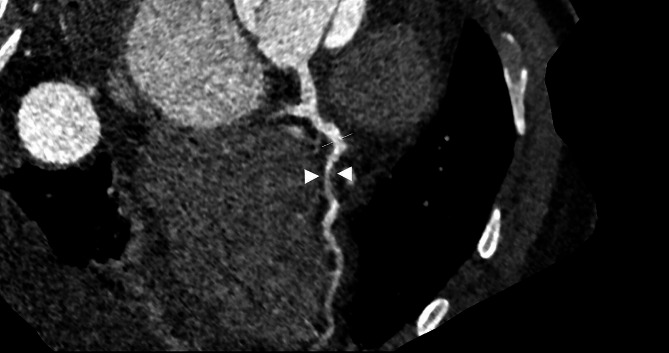
Reformatted image of CCTA demonstrating significant mid-LAD stenosis (white arrow heads)

**Fig. 2:**
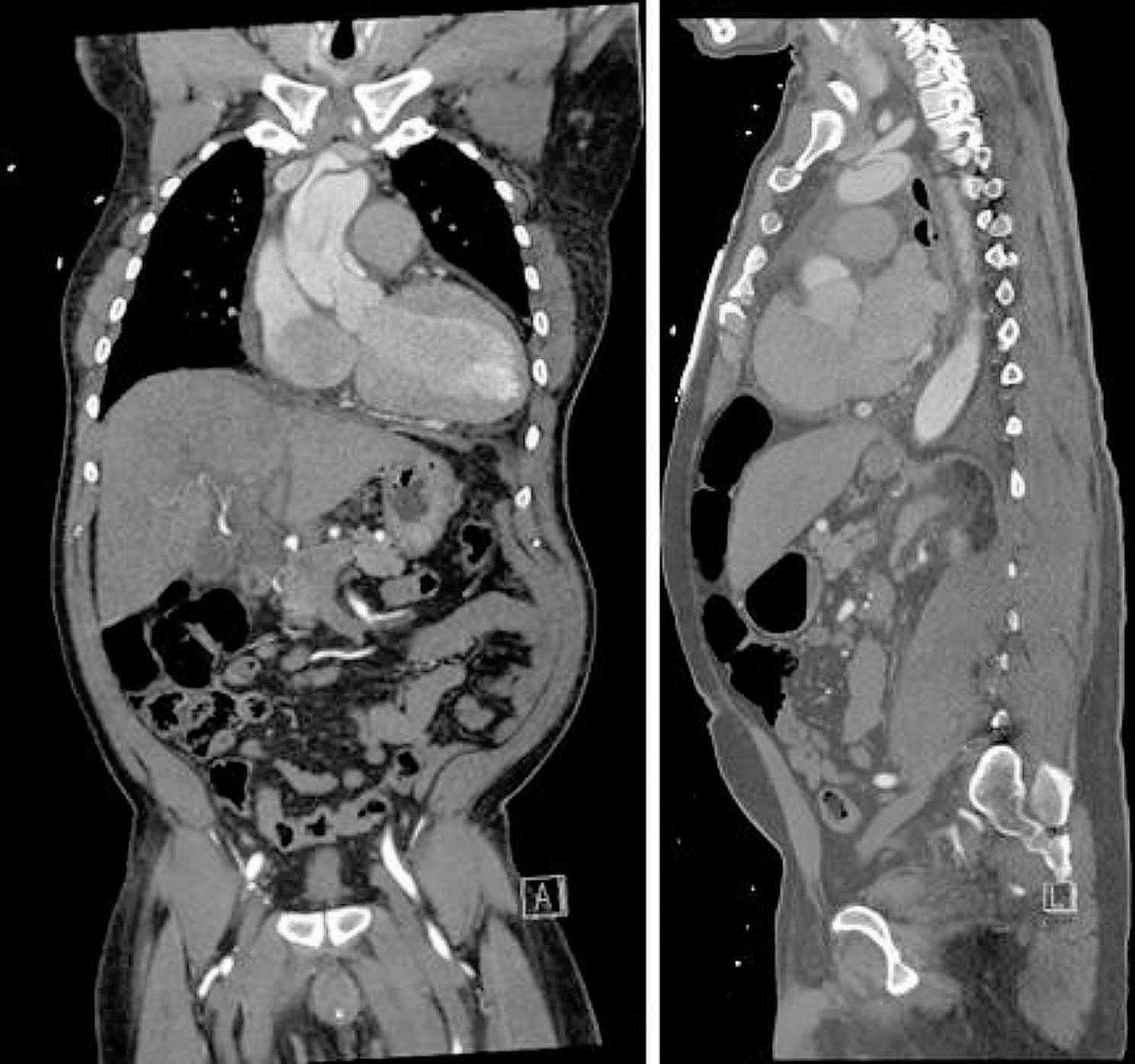
(**A**) Coronal view of Type A dissection flap in proximal aorta. (**B**) Sagittal view of non-dissected left subclavian artery coming off of dissected aortic arch

Given that the patient was approximately six days post-dissection, without malperfusion, contained rupture, pericardial or pleural effusions, with a well-controlled blood pressure on anti-impulse therapy, and had received five days of anticoagulation, the decision to delay definitive repair was made in order to let the apixaban metabolize and decrease the bleeding risk. While plausible that his heart failure and scar were related to coronary malperfusion secondary to his TAAD, emergent revascularization would have had limited benefit to the infarcted tissue considering the time from infarct. Given preserved GFR and no clinical renal malperfusion, CCTA was performed to assess the need for concomitant revascularization with a moderately obstructive proximal LAD lesion and complete occlusion of the mid-LAD after take-off of a large diagonal branch consistent with known prior infarction (Fig. 1), occlusive disease of the left circumflex artery as well as diagonal and obtuse marginal branches, and nonobstructive disease in the right coronary artery. Additionally, the CCTA demonstrated the dissection flap extending proximally to the sinotubular junction (STJ) (Fig. 2). Left heart catheterization was not performed, given concern for wire and catheter manipulation within a fresh dissection including navigating a multi-fenestrated flap.

Four days after his last apixaban dose, the patient was taken to the operating room for an ascending aorta hemiarch replacement with aortic valve resuspension, two-vessel CABG, and left atrial appendage clipping. Following sternotomy and pericardiotomy, the left internal mammary artery and saphenous vein graft conduits were harvested, and the patient was heparinized. Cardiopulmonary bypass (CPB) was instituted with central arterial and dual venous cannulation and left ventricular vent. The heart was arrested, and the patient was cooled to 18 °C. The aorta was transected at the STJ, where a circumferential tear was noted at the level of the STJ. The aortic valve was resuspended, and the intimomedial tear was plicated circumferentially to the true lumen.

After deep hypothermic circulatory arrest and the initiation of retrograde cerebral perfusion, as is routinely performed by the aortic surgeon at our institution, the diseased segment of the aorta was resected from the base of the innominate artery to the inner curvature of the proximal descending thoracic aorta. A 28-mm Gelweave graft was beveled and anastomosed to the native aorta to complete the distal anastomosis. An aortic cannula was placed into the side-arm for resumption of CPB. The proximal ascending aorta was resected to the level of the STJ, and the graft was sewn end-to-end to the STJ. The left atrial appendage was clipped with a 45 mm AtriClip (Atricure, West Chester, OH). The saphenous vein graft was anastomosed to the first obtuse marginal, and the left internal mammary artery was anastomosed to the LAD. The proximal vein graft was then anastomosed to the aortic graft. The patient was rewarmed, weaned off CPB, and chest closure was performed. There were no complications, and the patient was transported to the cardiovascular intensive care unit in stable condition.

Postoperatively, the patient was quickly extubated and weaned from vasopressors. The post-operative course was complicated by delirium and atrial fibrillation requiring direct current cardioversion. The patient was discharged home on post-op day 8; however, required readmission for volume overload which rapidly improved with diuresis and blood pressure control. Follow-up appointment ten days after readmission showed resolution of symptoms and normalization of blood pressure.

## Discussion and conclusions

This case highlights the successful surgical treatment of a patient with an acute TAAD and CAD via concomitant aortic repair with a CABG. While unconventional in contemporary practice, the case exemplifies a situation in which it is appropriate to perform pre-operative CCTA in order to perform concomitant CABG with TAAD repair. Several factors contributed to the decision to delay immediate surgical repair of the TAAD alone.

First, if performed, the emergent surgery would have taken place 5-to-6 days from dissection, which is relatively late in cases of acute TAAD, suggesting relative stability. According to one study, time-related mortality rates associated with acute TAAD are typically quoted as 1% per hour for the first 48 h after occurrence and approximately 50% at three days. (3–4) Additionally, the patient displayed no signs of malperfusion, the complication which is associated with the greatest risk of major morbidity and mortality in acute TAAD patients [5]. Furthermore, the patient was on anticoagulant therapy, specifically apixaban, which further increases the risk of bleeding already associated with cardiovascular surgery due to cardiopulmonary bypass and heparinization [6]. Another requisite included well-controlled blood pressure on anti-impulse/negative inotropic therapy, with the target of delaying tearing, improving myocardial perfusion, and preventing rupture [7]. 

Aortic repair was delayed in order to assess for CAD. In such cases, CCTA is preferred over conventional angiography via left heart catheterization to work up for CAD, as aortic manipulation is limited in the former. In conclusion, pre-operative CCTA and, if necessary, subsequent concomitant CABG may be considered in a select group of acute TAAD patients who present subacutely, without malperfusion, with well-controlled blood pressure on anti-impulse/negative inotropic therapy, and on anticoagulative therapy.

## Data Availability

No datasets were generated or analysed during the current study.

## Abbreviations

TAAD: Type A aortic dissection
CAD: Coronary artery disease
CABG: Coronary artery bypass grafting
CCTA: Coronary computed tomography angiography
LAD: Left anterior descending
STJ: Sinotubular junction
CPB: Cardiopulmonary bypass
